# Home-based palliative care for adult cancer patients in Ibadan—a three year review

**DOI:** 10.3332/ecancer.2014.490

**Published:** 2014-12-11

**Authors:** NE Omoyeni, OA Soyannwo, OO Aikomo, OF Iken

**Affiliations:** Hospice and Palliative Care Unit, University College Hospital (UCH), PMB 5116, Ibadan, Oyo 200212, Nigeria

**Keywords:** home-based palliative care, services, benefits

## Abstract

Home-based palliative care is a recognised model of health service provision globally, but is just emerging in Nigeria. The aim of this study isto review the spectrum of adult cancer patients involved in home-based palliative care, the services provided, outcome and benefits.

**Methods:**

Records of all adult cancer patients seen on home-based palliative care between March 2009 and January 2013 by the hospice and palliative care unit, University College Hospital (UCH), Ibadan were reviewed. Their biographical data, days on programme, diagnosis, stage of disease, major complaint, pain score, other symptoms, services offered, number of home visits, follow-up, and outcomes were extracted, reviewed, and analysed. The data were analysed using SPSS version 16.0.

**Results:**

Sixty patients were enrolled during the study period: there were 20 (33.3%) males and 40 (66.7%) females out of a total of 787 patients. All of them reside within catchment area of the hospice. Breast and prostate cancer constitute 21.7% each, gastrointestinal 16.7%, liver 11.7%, and cervical cancer 10.0%. Homes were visited 1–23 times per person. Days on programme ranged from 9–1207 days (average: 286 days). Pain was reported by 52 (86.7%) with scores of 7 to10 in 26 (50.0%). Only eight (13.3%) were pain-free. Services offered included pain and other symptom control, counselling and training for carers at home, provision of funds and comfort packs, bereavement services. The cost of services was heavily subsidised by the Centre for Palliative Care, Nigeria (CPCN), a non-governmental organisation and UCH. Although all patients are now deceased, the compassionate care received at a subsidised cost was highly valued, as shown from the appreciative comments of relations and carers.

**Conclusion:**

Home-based palliative care provided at low cost was beneficial to patients and their families. More can be achieved through the training of more health professionals, increased funding, and increased public awareness of the services.

## Introduction

The World Health Organisation (WHO) defines home care as the provision of health services by formal and informal caregivers in the home in order to promote, restore, and maintain a person’s maximum level of comfort, function, and health including care towards a dignified death. Home care services can be classified into preventive, promotive, therapeutic, rehabilitative, long-term maintenance, and palliative care categories. The strength of this approach is the dignity and privacy it gives to the patient and their families to be cared for in the comfort of the patient’s home, giving psychological, social, and spiritual support. Bereavement support is also an essential component of palliative care [1].

Home-based care was conceived as a means of alleviating the strain on overburdened and under-resourced hospitals whilst providing better and more holistic care to patients living with life-limiting illnesses such as cancer and human immunodeficiency virus (HIV)/acquired immune deficiency syndrome (AIDS). Groups of home-based carers, often drawn from religious groups, would visit patients in their communities, providing them with palliative and spiritual care, and educate the patients’ families in how to care for people living with HIV/AIDS.

Home-based care was meant to be a support mechanism for the hospital system and people living with HIV/AIDS (PLWA) and their families; a way to empower communities to respond to the impact of HIV/AIDS themselves by supporting them through the process. Home-based care however is now a recognised model of service provision for palliative care globally. Formal provision of home-based palliative care services for patients with chronic illnesses was started in Ibadan,Oyo State in 2006 by volunteering members of the CPCN, a non- governmental not for profit organisation. The UCH, a federal government tertiary hospital, in collaboration with the CPCN group established a hospice and palliative care unit in the hospital in 2007 to provide inpatient, daycare, home-based care services, and bereavement support. Patients enrolled for home-based care reside within the catchment area of the hospice, defined to be within a 30 km radius of the hospice location in Ibadan. This ensures that patients can be seen during working hours and maximises limited available manpower and financial resources. The unit also provides training for health professionals and carers. This is the first of such units in Nigeria. The UCH palliative care programme provides inpatient care, outpatient care at the hospice, daycare for adults and children living with life-limiting illnesses, including but not limited to those with cancer.

The aim of this paper is to discuss the spectrum of adult cancer patients seen by the unit and services offered to them in their homes from March 2009 to January 2013.

## Methodology

All patients referred to the hospice and palliative care unit were fully enrolled and consent was obtained for home-based palliative care from the patients and their main carers. The first visit was undertaken in the company of a carer or relation accompanying the palliative care team of doctor and nurse from the unit.

Records of 787 adult patients who had been enrolled for palliative care were reviewed to extract data on the patients with life-limiting illnesses who gave consent for home-based palliative care. Their biographical data, days on programme, diagnosis, stage of disease, major complaints, pain scores, other symptoms, services offered, number of home visits, follow-up, and outcome were extracted and reviewed. Pain was assessed using the Numerical Rating Scale (NRS): 0 = No pain,10 = Worst possible pain. The days on programme were obtained from the day of referral to the palliative care unit to the day of demise of the patient. The data were analysed using SPSS version 16.0.

### Results

A total of 60 adult cancer patients were enrolled for home-based care during the study period: there were 20 (33.3%) males and 40 (66.7%) females out of a total of 787 patients enrolled for palliative care in the period under review. The other 727 patients were not on home-based care for reasons such as: living outside the catchment area of the hospice or not giving consent for home-based care. However they received palliative care rendered to them either on the hospital wards as inpatients or in the hospice as outpatients and were followed up while on the wards and also via telephone. The age of patients ranged from19–98 years. The age distribution is as shown in Table 1.

All the patients had advanced stages of cancer: with 3 (5%) patients at stage II, 7 (11.7%) at stage III, and 50 patients (83.3%) T stage IV. The cancer diagnosis of these patients is shown in Table 2, with breast and prostate cancer constituting the highest at 21.7% each.

Homes were visited 1–23 times per person, an average of once per week and apart from symptom control, comfort packs (consisting of items such as food and toiletries) were given as a form of support. These items were provided through the CPCN—a not for profit organisation—or from individual donations to the unit. Days on programme ranged from 9–1207 days (average: 286 days).

**Figure 1.**
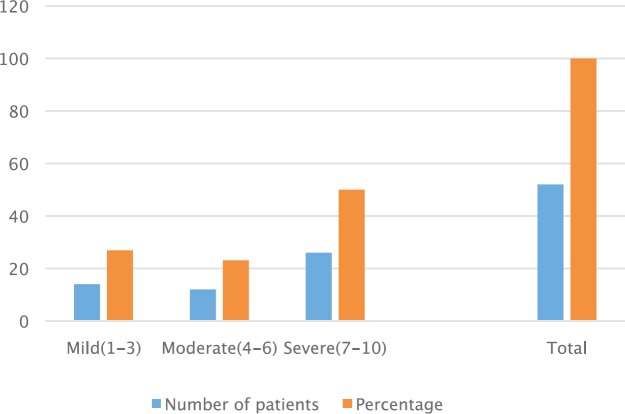
Pain scores of patients.

Pain was the major complaint in most patients. Out of the 60 enrolled patients, 52 (86.3%) had complaints of pain from multiple sites including primary and metastatic cancer sites and the scores on the NRS are as shown in Figure 1. Analgesics were administered based on the WHO analgesic ladder with oral liquid morphine being the strongest opioid analgesic and amitriptyline, the commonest adjuvant. Non-opioid analgesics included paracetamol, non-steroidal anti-inflammatory drugs (NSAIDs), weak opioids (tramadol, dihydrocodeine). All had pain scores less than two within three weeks of commencement of analgesics.

Patients complained of other symptoms such as cough, weight loss, weakness, pedal oedema, inability to sleep and fatigue for which they were managed appropriately by the team.

Other services offered at patients’ homes included psychosocial counselling and training of carers on wound dressing, drug administration, safe patient handling, and care. Some funds were provided for indigent patients as well as the comfort packs. Physiotherapy services were provided for some of the patients by the physiotherapist in the palliative care team when required. Apart from visits, patients were also followed up via telephone calls and bereavement support was also offered after death.

Although all patients were dead by the end of the review, their families were appreciative of the palliative care provided at subsidised cost, as reflected in their comments shown in Table 3.

## Discussion

Cancer remains one of the leading causes of death in developing countries. It has been reported that 650,000 people of an estimated 965 million are diagnosed with cancer yearly and that the lifetime risk of a woman dying from cancer in Africa is two times higher than in developed countries [2].

In this study, breast cancer was the commonest in women (21.7%) while prostate cancer was the leading cancer (21.7%) in men. This correlates with a previous review in Nigeria [3]. Prostate cancer was found to be the commonest cancer in males in Nigeria. It accounts for 6.1–19.5% of all cancers with a rising incidence [3, 4].

Home-based care has been practised informally by religious groups and individuals over the years with very little or no documentation. It however, remains an integral form of service provision for palliative care all over the world. The documented successes and benefits of these programmes include: enhancing the quality of life, easing the pressure on the few health facilities; alleviation of families’ lack of essentials; stigma reduction, and high levels of behaviour change.

Formal provision of home-based care in Nigeria is a relatively new concept and though it is still evolving, it has improved since 2007 when the UCH unit was established. As in the report by Parkins [2], all the patients referred to our palliative care unit had advanced stages of cancer with 80% at stage III and IV of the disease with poor outcomes.This may be because of the fact that most patients in sub-Saharan Africa present late to the hospital when they are ill and also the reluctance of primary physicians to refer patients early for palliative care. The former has been attributed among other reasons to the fear of the unknown, high prevalence of herbalists and religious houses, and ignorance. A study by Onyeka *et al* showed that 60–70% of patients with cancer present late in Nigeria. Many of the patients attribute their cancer as caused by spiritual forces. As many cancer patients die even after receiving conventional western medical treatment, other cancer patients become discouraged from seeking western medicine, and therefore resort to complementary and alternative medicine (CAM) treatments, prayers, or faith to obtain healing [5, 6].

Homes were visited between 1–23 times per person. This was usually determined by the clinical state of the patient at referral and the receptiveness of the family towards receiving the team. As part of the advocacy and getting patients families involved, a member of the family is asked to lead the team to the home of the patient for the first home visit. The costs of the visits are heavily subsidised by CPCN through fund raising to cover provision of funds and materials for the comfort packs, functional vehicle, and driver for transportation. Services are also rendered by volunteer members of CPCN who are mostly health professionals with palliative care skills. Comfort packs were given to indigent patients to reduce the cost of care and unnecessary suffering. The content of such packs is tailored to the possible needs of patients in a given locality and include pain medications, food items, toiletries, adult diapers, and sterile wound dressing materials to meet the basic needs of the patient. The continued provision of these comfort packs however would require both international and local funding from charitable organisations and individuals.

The University College Hospital (UCH), provides paid medical and non-medical staffs: doctors, nurses, administrative staffs who run the hospice. The latter constitutes the larger expense. Subsidisation of costs also includes lower consultation fees charged at the hospice and palliative care unit (about 50% less) than in the other hospital clinics and lower costs for oral morphine which is prepared from powder from the hospital pharmacy (about 75% less than imported liquid morphine). These efforts assist most of the patients who are very indigent to even have access to home-based care at the end of life.

All patients with life limiting illnesses referred to the unit and evaluated as requiring palliative care were enrolled on the programme. There are no criteria that limit the enrolment of patients on the programme, e.g., six months or less to likely death, as practised in some developed countries. An interdisciplinary team approach, focused on the patient and family, with care provided by a team consisting of a physician, nurse, social worker, and other trained health professionals were employed for the home-based care.

Although the average number of days on programme was 286 days, there were patients who were referred on the day they died and not much could be achieved because of the constraints of time and the disposition of the family at such a sensitive time. This further reinforces the need for health education for people to present early at hospitals and for health professionals to involve the palliative care team early and not wait until the end.

Pain was the most distressing complaint and the major reason for patient referral to the unit. The numerical rating scale 0–10 was used in scoring pain. Patients both educated and uneducated found it easy to understand and their caregivers too. It was used to assess pain at initial contact and also to rate pain at subsequent visits. Although most patients reported moderate to severe pain on enrolment, relief of distressing pain was achieved in the majority of patients. Liquid oral morphine prepared at low cost at the UCH pharmacy was the only strong opioid analgesic available in the country. Stocking, supply, and distribution of opioid medications to facilities where they are needed is done by the federal government. The challenges previously encountered on opioid availability and accessibility are now being resolved through the collaboration of the government and the Global Access to Pain Relief Initiative [7]. Other services offered included control of other symptoms, such as cough, dyspnoea, fatigue, for which appropriate treatment measures were instituted.

Emotional and spiritual support was also offered for psychosocial issues by members of the team. Issues around financial support, care for the family, job security and dependence on family with resultant loss of income are often discussed with patients and their families since healthcare in Nigeria is mostly catered for through ‘out of pocket’ expenses. The involvement of trained chaplains, Imams, or other religious leaders in home-based care would provide a more comprehensive and holistic service for Nigerian patients who are very religious.

By integrating palliative care into curative care practices earlier in the disease trajectory, chronically ill patients nearing the end of life report improved satisfaction with care and demonstrate less acute care use resulting in lower costs of care. In addition, patients enrolled in the palliative care programme were more likely to die at home than in comparison to group patients [8]. Home-based palliative care services help reduce the symptom burden people may experience as a result of advanced illness, without increasing grief for family caregivers after the patient dies. A ‘good death’ in a developing country occurs when the dying person is being cared for at home, is free from pain or other distressing symptoms, feels no stigma, is at peace, and has their basic needs met without feeling dependent on others [9, 10].

Thus, patients who wish to die at home should have affordable home palliative care with bereavement support [11, 12].

The needs addressed through the home-based care are multifaceted and many more patients with life-limiting illnesses would benefit if this is made available in their communities [13].

There are challenges which include the reluctance of some patients to receive home-based care, which is possibly because of the secretive nature of the African setting and the fear of stigmatisation. There is also insufficient funding for the provision of these services. Another limitation is the inadequate number of health professionals trained in palliative care, it being an emerging specialty in Nigeria. There is also the need for trained informal caregivers to provide and support the provision of home-based care in the community. The CPCN and other stakeholders are involved in advocacy for inclusion of palliative care including the home-based care model in the national health care system.

Most of the families who received care had come to see the palliative care team as an extended part of their families and sometimes they even discuss and request support on funeral plans and legal issues which are normally reserved for family members only. In general, families were appreciative of the subsidised home-based palliative care services provided. This reduced the burden of taking patients to and fro from the hospital and the purchase of oral morphine and other pain medications at higher cost. One of the patient’s family had this to say: ‘we really appreciate the palliative care team for the several visits to our home to see our father, their words of advice and encouragement, the regular phone calls to know how he is doing. We appreciate their efforts’. A patient (Mr A) also had this to say: ‘I thank the palliative care team because they have really shown themselves to be caring indeed. In fact, I have been able to get relief of my pain as chronic as it was before I met them. I am relieved with the way they have constructed my pain relief plan’.

## Limitations of the study

This study was a retrospective notes-based study and efforts are currently being made to institute a prospective study using standardised tools and outcome measures.

## Conclusions

This study reviews an innovative model of home-based palliative care services offered to adult cancer patients and the first of such in Nigeria. It highlights the importance of such a service in reducing expenses, community involvement, and the use of volunteers in end of life care. While so much is being done to improve the quality of life of these patients, more can be achieved through the training of more health professionals including aspects of effective pain management, increased funding, and increased public awareness on the availability of these services.

## Figures and Tables

**Table 1.** Age distribution of patients in years.

Age	Number of patients	Percentage (%)
11–20	2	3.3
21–30	1	1.7
31–40	2	3.3
41–50	6	10.0
51–60	10	16.7
61–70	15	25.0
71–80	13	21.7
81–90	8	13.3
91–100	3	5.0
Total	60	100.0

**Table 2.** Cancer diagnosis of patients on home-based care.

Cancer Diagnosis	Number of patients	Percentage (%)
Liver cancer	7	11.7
Breast cancer	13	21.7
Prostate cancer	13	21.7
Head and neck cancer	2	3.3
Gastrointestinal cancer	10	16.7
Cervical cancer	6	10.0
Lung cancer	2	3.3
Ovarian cancer	1	1.7
Soft tissue sarcoma	2	3.3
Non-Hodgkin’s lymphoma	4	6.6
		
Total	60	100.0

**Table 3.** Comments by carers.

Comments	Frequency	Percentage
‘Grateful for your care and support’	29	48.3
‘Appreciate services at subsidised cost’	20	33.4
‘Very grateful to the team’	11	18.3
Total	60	100.0
